# Overcoming missing data in spatial metabolomics with machine learning imputation to accelerate downstream discovery

**DOI:** 10.1016/j.isci.2026.115203

**Published:** 2026-03-03

**Authors:** Tingze Feng, Yuhan Wang, Shaojun Pei, Qiuping Wang, Yirong Li, Jing Lv, Tian Xia, Di Chen, Hai-long Piao

**Affiliations:** 1 State Key Laboratory of Phytochemistry and Natural Medicines, Dalian Institute of Chemical Physics, Chinese Academy of Sciences, Dalian 116023, China; 2 Department of Thoracic Surgery, Cancer Hospital of Dalian University of Technology, Liaoning Cancer Hospital and Institute, Shenyang 110042, China; 3 Department of Biochemistry and Molecular Biology, School of Life Sciences, China Medical University, Shenyang 110122, China; 4 University of Chinese Academy of Sciences, Beijing 100049, China

**Keywords:** bioinformatics, omics, metabolomics, machine learning

## Abstract

Mass spectrometry imaging (MSI)-based spatial metabolomics exhibits extensive missing values; yet, practical guidance on how imputation choices affect both imputation accuracy and downstream spatial analyses remains limited. In this study, we evaluated eight imputation methods, including both existing approaches and a graph convolutional network (GCN)-based method specifically designed for spatial metabolomics data, to identify suitable approaches for spatial metabolomics. To enable comprehensive assessment, we developed an evaluation framework focusing on two objective criteria: (a) imputation accuracy and (b) preservation of spatial cluster structure. We assembled six benchmark datasets spanning mouse brain and liver, human kidney and stomach, and plant seed sections, and conducted controlled dropout simulations of missing values. Across both evaluation dimensions, including imputation accuracy and preservation of spatial cluster structure, RF ranked first overall, and GCN ranked second in both dimensions. Overall, this systematic, dual-perspective benchmark study provides guidance for selecting imputation strategies in spatial metabolomics research.

## Highlights

• Eight imputation methods are benchmarked to address missing data in spatial metabolomics
• Performance is assessed on both imputation accuracy and spatial clustering preservation
• Random Forest and GCN emerge as top performers across multiple tissues and species

## Graphical abstract

**Figure:**
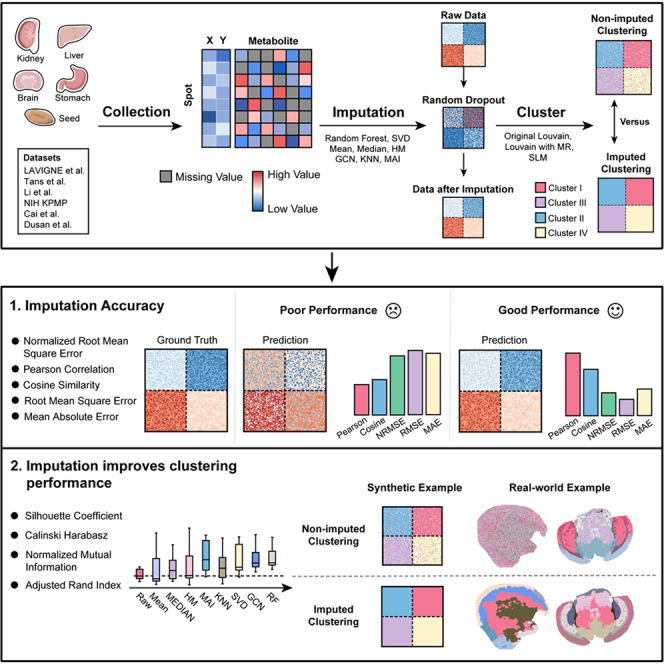

## Introduction

Spatial metabolomics (SM), a rapidly emerging research field, enables annotation, quantification, and analysis of metabolites and lipids within the spatial context of tissues, organs, and organisms.^1^^,^^2^ Revolutionized by technological advancements in mass spectrometry imaging (MSI) such as matrix assisted laser desorption/ionization (MALDI), desorption electrospray ionization (DESI), and secondary ion mass spectrometry (SIMS), spatial metabolomics have been widely applied to detect metabolic state of cells and their heterogeneity, reveal the regulatory role of metabolites and lipids in immune response and cancer, and create spatial maps of metabolic activity.^2^^,^^3^^,^^4^^,^^5^ Unlike bulk metabolomics, which relies on sample homogenization and loses spatial information, spatial metabolomics directly utilizes intact tissue slices to retain the spatial architecture of the tissue *in situ*, allowing for in-depth analysis.^6^^,^^7^ However, this preservation of tissue topology comes at a cost: spatial metabolite intensities generated by MSI suffer from high proportions of missing values.^8^ The prevalence of missing values in spatial metabolomics is notably higher than in LC/MS data, even when applied to the same tissue section, due to weak ionization efficiency, ionization suppression, and technical constraints specific to MSI acquisition.^6^^,^^8^^,^^9^ Several downstream analysis tools have been developed for processing spatial metabolomics data.^10^^,^^11^^,^^12^^,^^13^ Among them, only the *Multi-MSIProcessor* incorporated a selection of imputation methods.^12^ However, it lacked rigorous justification of imputation methods and could not provide the benchmarking framework, as in bulk metabolomics imputation benchmark studies, could guide users to choose the most appropriate algorithm for a given dataset or biological context.^14^ These limitations underscore the urgent need to identify robust imputation strategies in spatial metabolomics to enhance data completeness and ensure analytical reliability.

To address this, we present the first cross-species and multi-organ benchmark of eight imputation algorithms, including random forest (RF),^15^ k-nearest neighbors (kNNs),^16^^,^^17^ singular value decomposition (SVD),^18^^,^^19^ mean,^20^ median,^20^ mechanism-aware imputation (MAI),^21^ half of the minimum (HM),^20^ and graph convolutional network (GCN), applied to spatial metabolomics data from different analyzers. These methods spanned three methodological categories: traditional machine learning approaches (RF, SVD, kNN, and MAI), a deep learning model based on a customized GCN, and simple statistical imputations (mean, median, and HM). By evaluating these methods across 21 tissue-derived MSI slices from mouse, human, and plant tissues, we aim to comprehensively compare conventional and advanced algorithms in handling missingness within spatial metabolomics data. Moreover, we focused on two evaluation perspectives, one measuring the imputation accuracy and the other assessing the impact of imputation on downstream clustering analysis of the MSI data. Two imputation methods, RF and GCN, displayed better performance for imputing spatial metabolomics data. These results suggest that this benchmark study can provide valuable guidance for selecting suitable imputation strategies for spatial metabolomics data, thereby reducing the technique noise in MSI data and enhancing the detection of spatially coherent clusters.

## Results

### Overview of missingness in spatial metabolomics datasets

**Figure 1:**
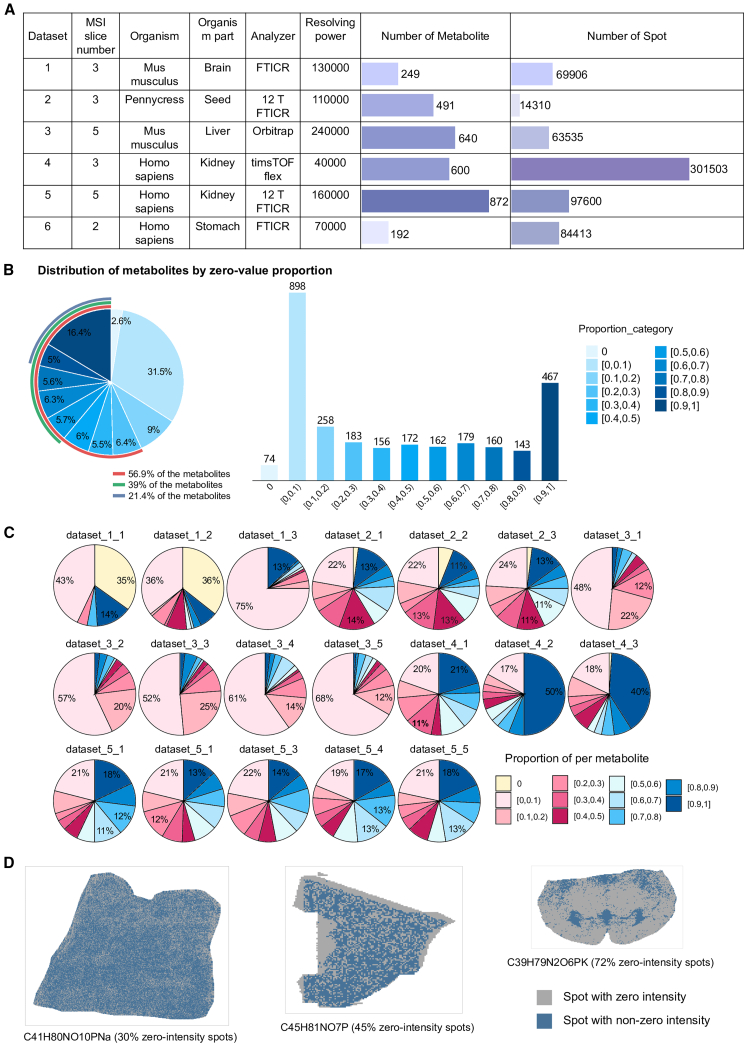
Landscape of missing values in spatial metabolomics (A) Summary of the benchmark datasets, including the number of sub-datasets, organism, tissue source, analyzer type, resolving power, and the number of spatial spots and annotated metabolites for each MSI slice. (B) Global distribution of per-metabolite zero-value proportions and counts across all collected datasets. Color encodes the zero-value proportion for each metabolite (0%–100% with zero intensity); each color level corresponds to the missingness of the metabolite. (C) Distribution of per-metabolite zero-value proportions in each MSI slice. As in (B), color represents the proportion of each zero intensity of metabolite within the individual sample. (D) Illustration of spatial distribution for metabolites with low, intermediate, and high zero ratios (three levels shown), demonstrating how zero values manifest in tissue space. See also Figure S1.

To establish a comprehensive benchmark for evaluating imputation methods in spatial metabolomics, we first characterized the extent of zero values across all collected datasets. In MSI spatial metabolomics, “zero values” mean a metabolite wasn’t detected (or had a signal below noise) in specific pixels, which is common due to low abundance, sample processing issues, or technical noise, leading to sparse data. Then, we applied eight algorithms—including seven established methods (RF, kNN, SVD, Mean, Median, MAI, HM) and one newly developed GCN-based approach—across six benchmark datasets at varying dropout rates (Figure 1A; Table 1 and method details). We simulated two metabolomics-relevant missingness mechanisms, missing completely at random (MCAR) and missing not at random (MNAR).^20^^,^^22^^,^^23^ Unexpected missing values arising from random errors or stochastic fluctuations during acquisition (e.g., incomplete derivatization or ionization) were treated as MCAR, whereas censored missing values due to being below the limit of quantification (LOQ) were treated as MNAR. For downstream clustering analysis, a separate realistic preprocessing strategy was used to simulate practical MSI data scenarios.

**Table 1:** Imputation methods for spatial metabolomics data

Methods	Language	Library
GCN	Python	Pandas, numpy, sklearn, torch, matplotlib
MAI	R	MAI^21^
RF	R	missForest^15^
KNN	R	impute^16^^,^^17^
SVD	R	pcaMethods^18^^,^^19^
Mean	R	https://github.com/WandeRum/MVI-evaluation
Median	R	https://github.com/WandeRum/MVI-evaluation
HM	R	https://github.com/WandeRum/MVI-evaluation

Subsequently, we collected 3,044 annotated metabolites from MATASPACE, each of which passed a spatial false discovery rate (FDR) threshold of ≤10%. This threshold reflects the probability of false-positive assignments in the context of MSI data, ensuring high-confidence spatial metabolite identification.^24^ As shown in Figure 1B, only 2.6% of these metabolites had non-zero intensities (excluding the two stomach slices). In contrast, 56.9% of the metabolites exhibited a zero rate greater than 20%, indicating that partial data loss is a widespread phenomenon. Importantly, 39% of metabolites had more than half of zero values, and 21.4% displayed proportions of zero exceeding 80% (Figure 1B). The patterns of metabolite zero values varied across the different datasets, reflecting the heterogeneity among datasets. Notably, all metabolites in Dataset_3, Dataset_4, and Dataset_5 showed zero values to varying extents, indicating that no metabolite was fully observed in these datasets (Figure 1C). We visualized the spatial distributions of representative metabolites with three levels of zero (low, mid, and high) across three sub-datasets (Figure 1D). The results reveal that zero values are distributed randomly, with no apparent association with specific organizational features. These results highlighted the intrinsic sparsity of spatial metabolomics data, even among high-confidence metabolites. The zero values may result from technical factors or detection limitations in these datasets, rather than true biological absence. Consequently, these zero values were treated as missing values to be imputed.

To systematically assess the performance of different imputation strategies in spatial metabolomics, we generated random missing values on these 19 slices, varying the proportion from 10% to 60% in increments of 10%. It is worth noting that we performed a filtering step prior to random dropout, retaining only those metabolites with fewer than 10% missing values in the original datasets, which ensured a reliable basis for evaluating index accuracy (Figure S1). The detailed filtering steps are provided in the STAR Methods section.

### Evaluation of eight imputation algorithms on spatial metabolomics data with simulated random dropout

**Figure 2:**
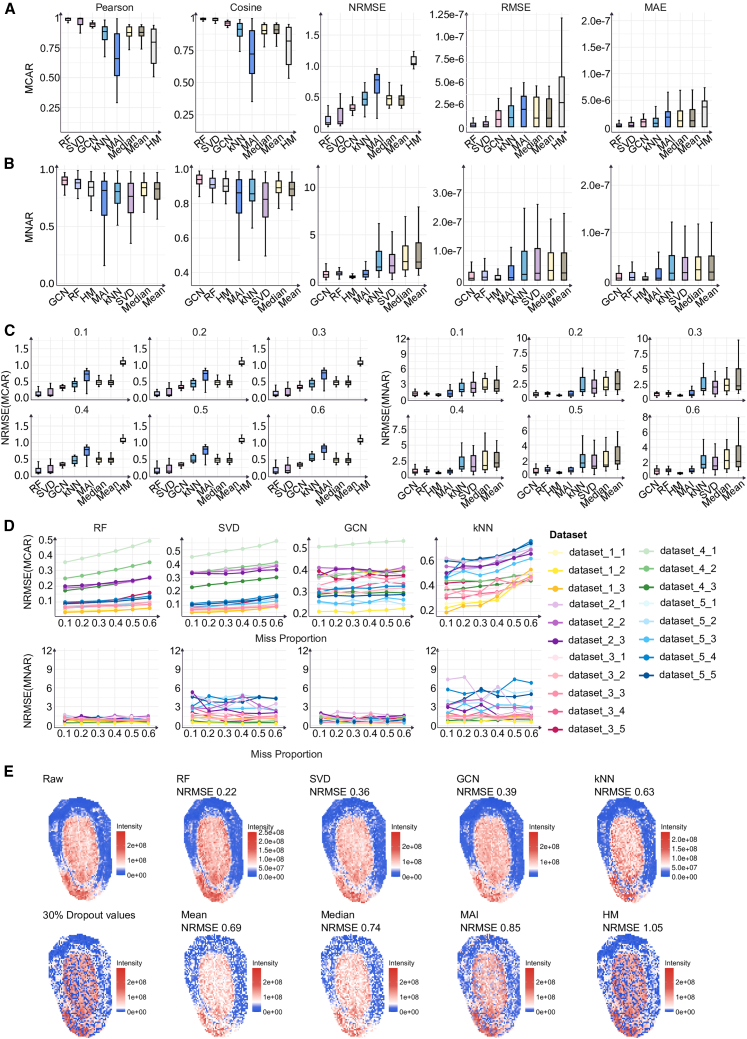
Overall performance and spatial reconstruction examples of different imputation methods under two missingness mechanisms (A and B) Summary of overall imputation performance under the MCAR (A) and MNAR (B) mechanisms. Boxplots show the distributions of Pearson correlation, cosine similarity, NRMSE, RMSE, and MAE (from left to right) across the 19 sub-datasets (aggregated over dropout settings). (C) Robustness comparison across dropout levels. Images on the left corresponds to MCAR and the right to MNAR; for each dropout proportion, boxplots display the distribution of NRMSE across the 19 sub-datasets for eight algorithms. In the boxplots (A–C), the central line indicates the median; the box boundaries represent the first and third quartiles (Q1 and Q3), and the whiskers extend to the most extreme data points within 1.5 × the interquartile range (IQR). (D) NRMSE trajectories of representative algorithms across datasets. The upper row shows MCAR and the lower row shows MNAR; curves depict NRMSE as a function of dropout proportion for RF, SVD, GCN, and kNN across the 19 sub-datasets, highlighting between-dataset heterogeneity and each algorithm’s sensitivity to increasing missingness. (E) Spatial reconstruction example under MCAR. Using metabolite C42H82NO8PK in Dataset_2_1 as an example, we show the original spatial intensity map (RAW), the observed map after 30% dropout, and the imputed reconstructions from eight algorithms; color encodes ion intensity, and all images use a unified color scale derived from the raw intensity range of this metabolite to facilitate comparison; the corresponding NRMSE is annotated for each reconstruction. See also Figures S1–S5.

We applied eight imputation methods (RF, SVD, GCN, kNN, mean, median, MAI, and HM) to 19 sub-datasets, and evaluated imputation performance under two missingness mechanisms, MCAR and MNAR, at six dropout levels (10%, 20%, 30%, 40%, 50%, and 60%). The imputation accuracy was evaluated using five metrics: NRMSE, RMSE, and MAE quantified error magnitude relative to the reference truth (pre-dropout reference), whereas the Pearson correlation coefficient and cosine similarity assessed linear concordance and directional consistency, respectively (Figures 2A and 2B). All accuracy metrics were computed by comparing the imputed matrices with the corresponding pre-dropout reference matrices. For methods requiring key hyperparameter selection (RF, kNN, SVD, and GCN), the five accuracy metrics reported and the example of spatial reconstructions are based on the hyperparameter configuration selected to minimize NRMSE under the corresponding dataset and missingness setting; specifically, after computing NRMSE for different hyperparameter combinations, we chose the configuration with the lowest NRMSE and reported all evaluation metrics (including NRMSE) under that configuration to enable fair comparison with other algorithms.

Under the MCAR setting, RF achieved the best overall performance (lower NRMSE, RMSE, and MAE, higher Pearson and cosine scores) across metrics and across dropout rates, followed by SVD and GCN (Figure 2A; left, Figure 2C). Line plot of NRMSE further showed that RF and SVD gradually degraded as dropout rates increased in some datasets (upper, Figure 2D). This fluctuation was more evident in datasets with large differences in the number of spatial spots (e.g., Dataset_4 with the most spots and Dataset_2 with the fewest). In contrast, GCN exhibited more stable NRMSE across different spot scales (upper, Figure 2D), suggesting lower sensitivity to spot number. Notably, although GCN was more stable, its overall error (NRMSE, RMSE, MAE) under MCAR remained higher than that of RF and SVD, highlighting a trade-off between stability and optimal accuracy. kNN and MAI exhibited markedly increased NRMSE at higher dropout rates (≥40%), indicating weaker robustness in high-missingness scenarios (upper, Figures 2D and S4A).

Under the MNAR setting, GCN achieved the best overall performance on NRMSE, RMSE, Pearson correlation, and cosine similarity, showing the lowest median NRMSE and RMSE and the highest median Pearson and cosine similarity (Figure 2B). In addition, HM showed favorable results for three metrics (NRMSE, RMSE, and MAE), which can largely be attributed to the alignment between the missingness generation scheme—where lower values were more likely to be missing—and HM’s underlying assumption that missing entries should be imputed with relatively low values. As dropout increased, the dispersion of NRMSE and MAE across datasets generally widened (right, Figures 2C and S3B), indicating stronger dataset heterogeneity under MNAR. For example, under mean imputation, the NRMSE of Dataset_2_1 increased from 3.94 at dropout = 0.1 to 7.88 at dropout = 0.6, whereas the NRMSE of Dataset_4_3 increased only slightly from 0.91 to 1.03. Line plots of NRMSE also showed that GCN and RF were relatively less affected by increasing missingness, whereas kNN degraded more substantially (lower, Figure 2D). Moreover, at dropout = 0.1, MAI performed poorly in the Dataset_2 sub-datasets with fewer spatial spots, suggesting that MAI may be more sensitive to spot scale (Figure S4B).

To provide a more intuitive and spatially resolved comparison of the eight methods, under the MCAR setting, we selected one metabolite from each of two sub-datasets, randomly masked 30% of its values, and visualized the corresponding imputations. As shown in Figure S5, we present metabolite C45H78NO7PNa from Dataset_1_1 (mouse brain tissue), while Figure 2E shows metabolite C42H82NO8PK from Dataset_2_1 (seed tissue). RF, SVD, and GCN more effectively reconstructed the original intensity distributions, whereas the other methods failed to recover the pre-dropout patterns. This contrast was more pronounced in the seed tissue section (Figure 2E). Notably, the spatial patterns produced by mean, median, MAI, and HM closely resembled the post-dropout observations, suggesting limited capacity to restore biologically meaningful variation.

### Impact of imputation on downstream clustering performance

Building upon the previous evaluation of imputation accuracy, we next investigated how imputation affects downstream spatial clustering, which was an essential analytical step in spatial metabolomics for delineating tissue-specific regions. During the imputation for samples, a different filtering criterion was applied: only metabolites with more than 80% missing values were excluded to preserve as much biological variability as possible and to better approximate the conditions of spatial clustering for real-world spatial metabolomics applications. Four evaluation metrics, the silhouette coefficient, the Calinski-Harabasz index, NMI, and ARI, were employed to assess the clustering performance. To ensure a comprehensive estimation, we examined clustering results under multiple clustering resolution parameters and across the performance of imputed data compared to that of the raw data. The details of the data processing strategy are presented in the method details section.

**Figure 3:**
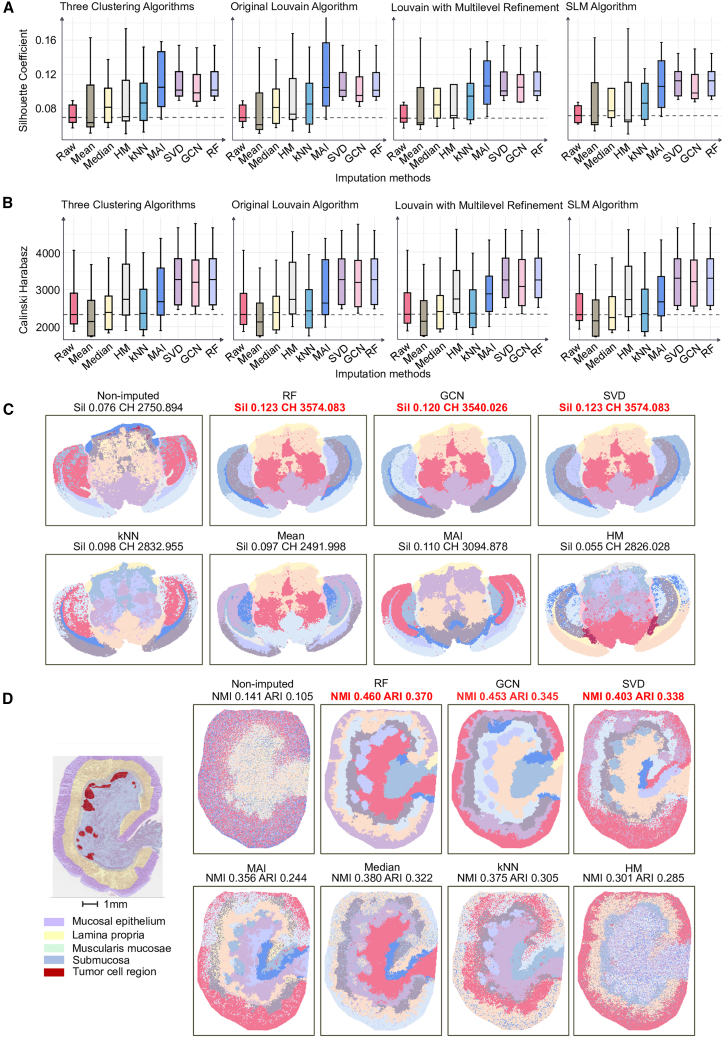
Evaluation of imputation effects on the performance of downstream clustering (A) Dataset_1_2’s silhouette coefficient across three cluster algorithms and nine resolution settings (0.1, 0.2, 0.3, 0.4, 0.5, 0.6, 0.7, 0.8, and 0.9). For each imputation method, clustering analysis was performed on the after-imputation matrix. Boxplots summarized the performance indexes over the nine resolutions; the dashed horizontal line indicated the median index of the non-imputed baseline. The first boxplots pooled results across all three cluster algorithms and all resolutions; the subsequent three boxplots report, for each clustering algorithm separately, the distribution of scores across the nine resolutions. Higher values indicated more compact and well-separated clusters. (B) Calinski-Harabasz index of Dataset_1_2 evaluated under the same design as in (A): boxplots over the nine resolutions per method, points for individual resolutions (0.1–0.9), and a dashed line marking the raw-data median reference. Higher values indicate stronger between-cluster separation relative to within-cluster dispersion. In the boxplots (A and B), the central line indicates the median; the box boundaries represent the first and third quartiles (Q1 and Q3), and the whiskers extend to the most extreme data points within 1.5 × the IQR. (C) Visualization of spatial clustering for raw data and seven imputation methods (algorithms labeled), shown at clustering resolution = 0.4 using the SLM cluster algorithm. Colors denote cluster identities in the spatial spot coordinate. All clustering analyses were performed on the pre-processed data (metabolites with >80% missingness removed), using identical spatial coordinates downloaded from MATASPACE. The corresponding silhouette coefficient and Calinski-Harabasz index were annotated for each spatial clustering. Sil, silhouette coefficient; CH, Calinski-Harabasz. The two values highlighted in red corresponded to metrics that ranked within the top three among the eight algorithms. (D) Extended assessment of the impact of imputation on spatial clustering in the human gastric cancer sample (gc_1). Images on the left shows the pathologist-annotated region map on the H&E-stained tissue section (Scale bars, 1 mm), where purple shading denotes mucosal epithelium, yellow denotes lamina propria, green denotes muscularis mucosae, red denotes the tumor cell region, and blue denotes submucosa. Images on the right showed spatial clustering results obtained without imputation and after imputation using seven different methods; all methods were constrained to produce nine clusters. For each spatial clustering result, the corresponding NMI and ARI values were labeled. The two values highlighted in red corresponded to metrics that ranked within the top three among the eight cluster results. See also Figures S6 and S7.

RF significantly improved clustering performance in both sub-datasets (Dataset_1_2 and Dataset_2_1). Compared with the raw, non-imputed data, RF achieved the best overall results in terms of the silhouette Coefficient and the Calinski-Harabasz index, which indicated robust preservation of inter-cluster separation and global spatial structure (Figures 3A, 3B, S6A, and S6B). GCN also showed strong performance in both datasets, and it ranked among the top three for both metrics, with third place in Dataset_1_2 and second place in Dataset_2_1. In contrast, SVD and MAI showed limited consistency: although each performed relatively well in either Dataset_1_2 or Dataset_2_1, they did not maintain comparable performance in the other dataset (Figures 3B and S6B). In particular, SVD performance dropped markedly in Dataset_2_1, with a median Calinski-Harabasz index close to that of the non-imputed data, suggesting higher sensitivity to the number of spatial spots and insufficient robustness in maintaining compact, well-separated cluster structures. Additionally, we observed that the benefit of imputation for improving clustering quality was more pronounced in datasets with a larger number of spatial spots. As illustrated in Figure 3C, the clustering results based on raw data appeared fragmented, with diffuse cluster boundaries and substantial noise, which was likely attributable to the high proportion of missing values. After imputation, particularly with the RF, SVD, and GCN algorithms, the spatial delineation of clusters displayed fewer noisy regions and improved separation between clusters. However, these improvements in spatial clustering quality following imputation were less pronounced in samples with fewer spatial spots (Figure S6C).

In addition, to further validate the robustness of different imputation algorithms in annotated tissue samples and to assess whether imputation more accurately recovered known spatial tissue architecture, we performed spatial clustering on two gastric cancer slices with H&E-annotated regions and quantified the impact of different imputation strategies on clustering accuracy using NMI and ARI (Figures 3D and S6). The results showed that, in both sections, clustering based on raw non-imputed data failed to form coherent spatial domains and exhibited prominent noisy regions. In contrast, clustering after RF imputation achieved the best overall performance in both slices. In the gc_1 slice, RF, GCN, and SVD identified the tumor cell region. RF and GCN further delineated mucosal epithelium, submucosa, and lamina propria, whereas the other imputation methods generally separated these regions but produced more fragmented boundaries and more pronounced noise (Figure 3D). In the gc_2 slice, aside from the raw non-imputed result and the HM-imputed result that showed substantial noise, the remaining imputation methods differentiated the mucosa, tumor cell region, and submucosa to some extent. Notably, all methods failed to consistently identify the lymphoid follicle region, which was likely due to the relatively small predefined number of clusters. Taken together, RF and GCN maintained good clustering performance in samples with different numbers of spatial spots, with approximately 38,000 spots in gc_1 and approximately 28,000 spots in gc_2, which indicated that both methods showed strong robustness and adaptability to missing data in complex tissue environments.

### Final evaluation: Overall performance comparison across imputation methods

**Figure 4:**
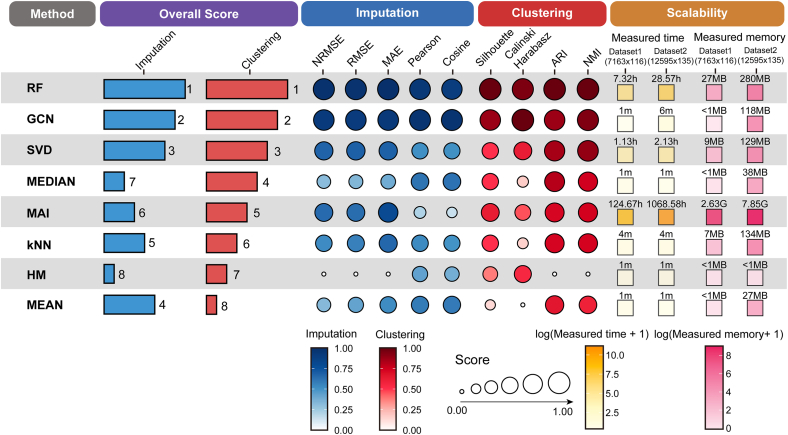
Overall ranking of the eight imputation methods based on combined evaluation metrics Individual metric scores and aggregated performance scores are depicted using circles and bars, respectively. Circles and bars representing the imputation accuracy and the preservation of spatial cluster power were colored in blue and red, respectively. The intensity of color corresponded to the overall ranking, with darker shades indicating superior performance. The scalability section compared the computational cost of each imputation algorithm on two representative datasets, including measured runtime (measured time) and peak memory usage (measured memory). Dataset1 had a matrix size of 7,163 × 116, and Dataset2 had a matrix size of 12,595 × 135 (spots × metabolites). The numbers in each square indicated the measured runtime (m/h) and memory (MB) for the corresponding dataset, and the color encoded the log-transformed values, namely log (measured time +1) and log (measured memory +1) (see color bars), where “+1” avoided log (0) and compressed the dynamic range for cross-scale comparison.

To comprehensively evaluate and compare the overall performance of the imputation methods, we integrated two key evaluation dimensions to construct an overall performance ranking, including imputation accuracy and preservation of spatial clustering structure (see method details). As shown in Figure 4, RF consistently performed best in both dimensions, followed by GCN and SVD. We further evaluated scalability by measuring runtime and peak memory usage on two representative datasets with different matrix sizes. The results showed that simple statistical methods, such as mean and HM, required the least computational resources. RF generally incurred the second-highest runtime and memory costs overall. GCN maintained strong performance while achieving relatively better computational efficiency, which reflected a favorable balance between performance and computational cost. MAI required substantially longer runtime on both datasets and exhibited the highest peak memory usage. In contrast, simple statistical methods such as mean, median, and HM ranked lower in both dimensions. Notably, although MAI was designed to first classify the missingness mechanism and then apply mechanism-specific imputation, it did not show a clear advantage in either imputation accuracy or spatial structure preservation under our datasets and evaluation framework. For the imputation accuracy dimension, RF ranked first on all three error-based metrics, including NRMSE, RMSE, and MAE, which indicated higher reconstruction precision in recovering metabolite intensity magnitudes. GCN ranked first on the Pearson correlation coefficient and cosine similarity, which suggested that it better preserved global trends and directional consistency. For the preservation of spatial clustering structure, RF ranked first on the silhouette coefficient, ARI, and NMI, which indicated stronger within-cluster compactness and higher agreement with the reference tissue structure. GCN ranked first on the Calinski-Harabasz index, which reflected an advantage in enhancing between-cluster separation and overall structural clarity. These results, derived from multiple complementary metrics, systematically characterized the trade-off between intensity reconstruction accuracy and spatial structure fidelity across imputation strategies and provided more targeted guidance for method selection in different application scenarios.

## Discussion

This study is the first comprehensive benchmark of eight imputation methods for MSI-based spatial metabolomics, using a dual perspective that evaluates both numerical accuracy and the preservation of spatial cluster structure (Figure 4). We assembled six benchmark datasets from METASPACE, spanning mouse brain and liver, human kidney, and plant seed sections, and performed controlled dropout simulations across multiple missingness levels to systematically evaluate method performance. The results indicated that RF was the most recommended method for imputation in spatial metabolomics, ranking first overall in both imputation accuracy and preservation of spatial clustering structure. GCN ranked second in both dimensions, suggesting that it provided consistently strong performance while effectively leveraging spatial structure information. Considering practical computational cost, we recommended prioritizing RF when researchers had sufficient runtime budget. When researchers required faster execution, we recommended using GCN as an alternative to RF. This systematic, dual-perspective benchmark provided a reproducible evaluation framework and actionable guidance that could improve the reliability of missing-value handling in spatial metabolomics and support method selection in future research.

Our results aligned with evidence from bulk metabolomics analysis showing that RF was resilient to missing values completely at random in metabolomics data.^20^ Moreover, our study also extended recent findings. In addition to being applied in the field of spatial transcriptomics for clustering,^25^^,^^26^ GCN can also be repurposed for spatial metabolomics data imputation. Through the use of the spatial adjacency matrix, GCN smoothed missing values over meaningful neighborhood spots, thereby improving downstream inference of spatial metabolomics. In contrast, RF exploited nonlinear metabolite-metabolite dependencies without requiring explicit spatial priors.^15^ We observed that some methods showed unstable performance under high missingness or sparse spatial sampling. For example, kNN deteriorated more noticeably as missingness increased, and SVD was more likely to be affected by its global low-rank assumption when spot counts were low or spatial coverage was heterogeneous, which could yield negative imputed values that required additional zero-clipping.

### Limitations of the study

Due to current limitations of MSI detection technology, it remained difficult to strictly distinguish “true biological absence” (for example, signals that were genuinely absent or below biological background) from “technical non-detection” (for example, values below the detection limit or missingness induced by noise) in real datasets. Future work should more precisely characterize missingness mechanisms and improve the biological interpretability of imputation by integrating detection-limit information, background-noise modeling, and more detailed experimental design to distinguish and model missing and zero values at a finer granularity. Although we aimed to cover diverse tissue types and mass analyzer platforms, the current benchmark still relied primarily on publicly available MSI data accessible through METASPACE, so full extrapolation to other platforms, more extreme resolution settings, or laboratory-specific acquisition and preprocessing workflows remained limited. Future work should aim to adapt algorithm parameters to the specific characteristics of each dataset, such as cluster resolution and the extent of missingness, to enhance both imputation accuracy and the spatial clusters identification. In parallel, assembling or generating spatial metabolomics datasets with pathologist-adjudicated, co-registered histopathological region annotations will be essential for enabling robust external validation. Additionally, hybrid frameworks that integrate the nonlinear modeling capabilities of RF with the spatial awareness of GCN may help mitigate optimistic bias and improve the reliability of performance estimates.^15^

## Resource availability

### Lead contact

Further information and requests for resources and reagents should be directed to and will be fulfilled by the lead contact, Hai-long Piao (hpiao@dicp.ac.cn).

### Materials availability

This study did not generate new unique reagents.

### Data and code availability

• This study analyzes existing, publicly available data, accessible at METASPACE platform (https://metaspace2020.org/).^27^ The ID numbers for each set of data at METASPACE can be found in STAR Methods.
• The code for benchmark analysis has been deposited at GitHub (https://github.com/SQZ-0000/Spatial_metabolomics_benchmark_code) and is publicly available as of the date of publication. The link is also listed in the key resources table.
• Any additional information required to reanalyze the data reported in this study is available from the lead contact upon request.

## Acknowledgments

We thank Dr. Chenxing Jin from the Department of Pathology, 10.13039/501100010108Zhongshan Hospital, 10.13039/501100003347Fudan University, Shanghai, China, for assisting with the histopathological annotation of the gastric cancer tissue slices. This study is supported by the 10.13039/501100001809National Natural Science Foundation of China grants (no. 32470832), 10.13039/501100018617Liaoning Revitalization Talents Program (XLYC2002035), Science and Technology Innovation Fund (Youth Science and Technology Star) of Dalian (no. 2021RQ009), Innovation program of Science and Research from the DICP, CAS (DICP I202414).

## Author contributions

T.Z., H.-l.P., and D.C. conceived and designed the project. T.Z. conducted the analysis and generated data, and T.Z., H.-l.P., and D.C. wrote the manuscript. Y.W., S.P., Q.W., Y.L., J.L., and T.X. contributed to data analysis and discussed the results. All authors read and approved the final manuscript.

## Declaration of interests

The authors declare no conflict of interest.

## Declaration of generative AI and AI-assisted technologies in the writing process

During the preparation of this work, the authors used ChatGPT5.2 in order to polish and refine the wording of the manuscript. After using this tool or service, the authors reviewed and edited the content as needed and take full responsibility for the content of the publication.

## STAR★Methods

### Key resources table

**Table:** 

REAGENT or RESOURCE	SOURCE	IDENTIFIER
**Deposited data**		

Spatial Metabolomics datasets	Alexandrov et al.^27^	https://metaspace2020.org/
Code for benchmark analysis	This paper	https://github.com/SQZ-0000/Spatial_metabolomics_benchmark_code

**Software and algorithms**		

MAI(v1.10.0)	Dekermanjian et al.^21^	https://github.com/KechrisLab/MAI
missForest(v1.5)	Stekhoven et al.^15^	https://github.com/stekhoven/missForest
impute(v1.78.0)	Trevor et al.^16^^,^^17^	https://bioconductor.org/packages/impute
pcaMethods(v1.96.0)	Stacklies et al.^18^^,^^19^	https://github.com/hredestig/pcamethods
Mean/Median/HM imputation	Wei et al.^20^	https://github.com/WandeRum/MVI-evaluation
GCN imputation	this paper	https://github.com/SQZ-0000/Spatial_metabolomics_benchmark_code
ggplot2(v3.4.2)	CRAN / Bioconductor	https://github.com/tidyverse/ggplot2
ggpubr(v0.4.0)	CRAN / Bioconductor	https://rpkgs.datanovia.com/ggpubr/

### Experimental model and study participant details

This study did not involve the recruitment of new experimental models or study participants. All analyses were conducted using publicly available datasets from METASPACE.^27^ No samples were excluded or reallocated based on sex, gender, ancestry, race, or ethnicity in the present study.

### Method details

#### Benchmark data sets for evaluating data imputation methods

Six benchmark datasets were applied to evaluate the data imputation methods. All datasets used in this study were downloaded from the METASPACE platform (https://metaspace2020.org/),^27^ comprising a total of 21 tissue slices. These include two datasets from Mus musculus tissue sections: one from brain tissue (three tissue sections, 249 metabolites, 69,909 spots, METASPACE: 2016-09-22_11h16m17s,2016-09-22_11h16m40s, 2016-09-22_11h16m33s) and one from liver tissue (five tissue sections, 640 metabolites, 63,535 spots, METASPACE: 2022-07-13_21h28m34s, 2022-07-13_21h29m14s, 2022-07-13_21h30m17s, 2022-07-13_21h30m44s, 2022-07-13_21h31m40s). In addition, two datasets were derived from human kidney tissue sections. One dataset includes three tissue sections, with 600 metabolites across 301,503 spots (METASPACE: 2023-01-13_15h22m08s, 2023-01-19_20h45m02s, 2023-01-19_20h59m24s), while the other contains five sections, comprising 872 metabolites with 97,600 spots (METASPACE: 2025-01-15_22h38m03s, 2025-01-15_22h38m30s, 2025-01-15_22h39m00s, 2025-01-15_22h40m18s, 2025-01-15_22h40m42s). One dataset includes two stomach cancer tissue slices, with 192 metabolites across 84,413 spots (METASPACE: 2023-11-27_04h08m51s, 2023-11-27_04h08m34s).^28^ Lastly, a dataset from pennycress seed sections was included, comprising three tissue sections, 491 metabolites, and 14,310 spots (METASPACE: 2024-04-13_01h19m39s, 2024-04-13_01h20m40s, 2024-04-13_01h21m21s).

#### Artificial missing value generation

To ensure the reliability and robustness of the evaluation metrics, preprocessing steps were applied to all datasets before the imputation experiments. First, spatial spots with zero intensity values across all metabolites were removed, as they do not provide informative biochemical signals. Second, we retained only those metabolites with a missing/zero value rate below 10%, to ensure that the majority of their values represent true observed intensities. This filtering strategy was adopted to establish a high-confidence reference for assessing imputation accuracy. Then, to systematically evaluate the performance of different imputation methods under varying levels of missingness, we constructed two missingness mechanisms commonly observed in metabolomics data: missing completely at random (MCAR) and missing not at random (MNAR). In MS-based metabolomics, missing values are generally categorized into three types: MNAR, missing at random (MAR), and MCAR. However, MAR and MCAR are often difficult to clearly distinguish, and some studies treated all MAR values as MCAR values.^29^ Therefore, we focused our simulations on MCAR and MNAR only. The construction of MACR and MNAR followed missingness-generation strategies commonly used in published work.^20^ MCAR generation: In each filtered dataset, we artificially introduced missing values into the filtered datasets at six dropout rates: 10%, 20%, 30%, 40%, 50%, and 60%. MNAR generation: In each filtered dataset, we first randomly selected the corresponding proportion of metabolites (10%, 20%, 30%, 40%, 50%, and 60%); for each selected metabolite, we randomly generated a truncation quantile threshold q∈[0.30,0.60] and replaced observations below its q^-th^ quantile with missing values to implement an MNAR mechanism in which lower-intensity values are more likely to be missing.

#### Data imputation methods for spatial metabolomics

Eight Imputation approaches were employed (Table 1). Seven of these are pre-existing methods that have been widely applied in metabolomics data,^23^ including RF,^15^ kNN,^16^^,^^17^ SVD,^18^^,^^19^ Mean,^20^ Median,^20^ MAI,^21^ and HM.^20^ Except for them, a GCN-based imputation method was also developed.

1. GCN imputation: We developed a customized graph-based autoencoder that integrated spatial neighborhood information with metabolite intensity profiles. This method was performed utilizing the PyTorch framework. The detailed procedures regarding data preprocessing, graph construction, network architecture, and model training are described as follows.

##### Data preprocessing and graph construction

Raw metabolite intensity matrices were log-transformed and then scaled to the interval [0, 1] using min-max normalization. A binary mask was generated to label observed and missing values, ensuring that the reconstruction loss during training was from observed values. The spatial coordinates (*x, y*) of each spot were used to construct a kNN graph (*k* = 8 by default), in which each node represented a spatial spot, and edges connected the eight closest neighbors. The adjacency matrix was then symmetrized, and the corresponding edge list was provided to the GCN layers for message passing.

##### Model architecture

We designed an autoencoder architecture with graph convolutional layers (GCNConv) serving as both encoder and decoder. The encoder consisted of three sequential GCNConv layers, which reduced the input feature dimension (number of metabolites) to a compact latent embedding. In our configuration, an input layer of dimension *d* (the number of metabolites) was mapped through hidden layers of [32, 16] to a bottleneck embedding of 16 dimensions. Each layer was followed by batch normalization, rectified linear unit (ReLU) activation, and dropout (rate = 0.2) to prevent overfitting. The decoder mirrored this architecture, progressively reconstructing the input dimension via layers of size 64 and 128, followed by a GCNConv layer that projected back to the original metabolite dimension. Finally, a sigmoid activation function constrained reconstructed values to the range [0, 1].

##### Model training

The model was trained for a maximum of 1000 epochs using mean squared error (MSE) as the reconstruction loss function. Model parameters were optimized using the Adam optimizer (learning rate = 10^-3^, weight decay = 10^-5^). To improve generalization and prevent overfitting, an early stopping strategy was employed (patience = 20 epochs).

2. RF imputation: RF imputation constructed predictive models by iteratively treating each variable with missing values as a target and using the remaining variables as predictors. We performed RF imputation using the R package *missForest.*
3. SVD Imputation: This method initialized missing values with zero and estimates them iteratively as a linear combination of the top k principal components, until convergence. Before imputation, the data matrix was scaled and centralized. We performed SVD imputation through the R package *pcaMethods.*
4. kNN Imputation: kNN identified the k most similar instances using Euclidean distance and imputes missing values by averaging the corresponding non-missing values of the neighbors, which was initially developed for microarray gene expression data. This method was conducted through the R package *impute.*
5. MAI: MAI developed a two-stage framework. First, it applied a Random Forest model to infer the underlying missingness mechanism for each data point. Then, the algorithm assigns each missing value to the most appropriate imputation method tailored to the predicted mechanism. This method was conducted through the R package *MAI.* Because MAI exceeded the predefined runtime limit (5 days) on the full Dataset_4 under the missingness mechanism, we did not record its evaluation metrics for Dataset_4.
6. Mean Imputation: This method replaced missing values with the mean of the non-missing values across all samples.
7. Median Imputation: Similar to the Mean imputation, this approach used the median of the non-missing values to fill in each missing value.
8. HM Imputation: This method replaced missing entries with half of the minimum non-missing value for each variable, a simple yet commonly used approach in metabolomics studies.

#### Sensitivity analysis and selection of key hyperparameters for imputation algorithms

To examine whether key parameter settings affect imputation accuracy under two missingness mechanisms (MCAR and MNAR), we systematically tested the major hyperparameters of four imputation algorithms and compared the performance of different parameter configurations across all datasets. For RF imputation, we evaluated *ntree* set as 100,200,300,500, and 800 across all datasets. For kNN imputation, we tested different numbers of neighbors, *k* set as 5,10,15,20, and 30. For SVD imputation, we varied the rank (number of components) with values 5,10,15,20, and 30. During rank tuning for SVD, we observed that SVD produced negative imputed values in some datasets. Given the non-negative nature of mass spectrometry intensity data, we clipped negative values to zero and compared NRMSE before and after clipping. The results showed that zero clipping reduced NRMSE for some datasets under specific dropout settings, so we added zero clipping of negative values as an additional post-processing step for SVD imputation (Figure S9). This artifact likely arises from the mathematical formulation of SVD-based imputation, where missing values are estimated through low-rank approximation using a linear combination of singular vectors and values.^17^^,^^18^^,^^19^^,^^30^^,^^31^ As these combinations are unconstrained, negative estimates can occur in datasets with limited spatial coverage or high missingness.^17^^,^^30^^,^^32^^,^^33^^,^^34^^,^^35^ The results indicated that changing parameters for RF and kNN had little impact on imputation accuracy, whereas for SVD, setting the rank to 10 yielded a slight improvement. For GCN imputation, we further conducted ablation experiments to identify parameter settings that are more stable for downstream analyses. We focused on three hyperparameters: the number of neighbors *k* used to construct the adjacency matrix, *the hidden-layer architecture (hidden_dims)*, and *the embedding dimension (embedding)*. The ablation study proceeded sequentially: we first examined the effect of *k* with *k* = 4,6,8,10, and 12, while fixing *hidden_dims* = (24, 16, 8) and *embedding* = 8. Under this setting, we compared imputation performance across 19 datasets at different dropout rates and proceeded to the next step after selecting *k* = 8. Next, with *k* = 8 and *embedding* = 8 fixed, we evaluated different hidden-layer configurations: (16), (32, 16), (24, 16, 8), (32, 24, 16, 8), and (64, 32, 16, 8). Finally, with *k* = 8 and *hidden_dims* = (32, 16) fixed, we further compared different embedding dimensions: (4, 8, 16, 32, 64). Parameter selection was based on the NRMSE values across datasets under both MCAR and MNAR mechanisms (comparing performance across dropout rates) for each parameter setting. Based on these results, in the subsequent clustering analysis of gastric cancer samples, we used default settings for RF and kNN, fixed the SVD rank at 10, and set the GCN hyperparameters to *k* = 8, *hidden_dims* = (32, 16), and *embedding* = 16.

#### Downstream clustering analysis

For downstream clustering analysis of MSI data, we applied a distinct preprocessing strategy to simulate a realistic clustering scenario. Specifically, after removing spatial spots where metabolite intensities were zero across all variables, we further excluded metabolites with more than 80% missing values. Following this two-step filtering process, eight imputation algorithms were applied to estimate the missing values. Clustering was then performed using the imputed metabolite matrices, and results were compared with those derived from the preprocessed but non-imputed datasets. Clustering analysis was performed using the R package *Seurat*.

#### Evaluation index for eight imputation methods

For imputation accuracy, we applied eight distinct imputation algorithms to six dropout rates to recover the missing values for each dataset. The accuracy of each imputation method was then quantitatively assessed by comparing the imputed matrices against the corresponding original (pre-dropout) data using the following five evaluation indices:

1. Normalized Root Mean Square Error (NRMSE): NRMSE quantifies the deviation between imputed and true values, normalized by the standard deviation of the original data, making it a scale-independent measure of imputation accuracy. Lower NRMSE values indicate more accurate imputation performance, with values closer to zero reflecting minimal reconstruction error.^20^^,^^36^^,^^37^
2. Pearson correlation coefficient: The Pearson correlation coefficient evaluates the linear relationship between imputed and true values, providing insight into how well the imputation preserves global data trends.^38^^,^^39^ Higher Pearson values (closer to 1) suggest stronger agreement and greater preservation of the original data structure.
3. Cosine similarity: Cosine similarity measures the angular similarity between the imputed and true data vectors, independent of magnitude, capturing the consistency in data orientation.^40^ A value approaching 1 indicates a high degree of directional alignment, signifying reliable structural reconstruction.
4. Root Mean Square Error (RMSE): RMSE measures the reconstruction error between imputed and true values by taking the square root of the mean squared differences.^41^ Lower RMSE indicates more accurate imputation.
5. Mean Absolute Error (MAE): MAE quantifies the average absolute errors between imputed and true values, providing an interpretable measure of typical error magnitude that is less sensitive to large outliers than RMSE.^42^ Lower MAE indicates better performance.

For downstream clustering analysis, we evaluated the impact of imputation or non-imputation on spatial clustering using the following four evaluation indices:

1. Silhouette coefficient: The Silhouette coefficient assessed clustering validity by comparing intra-cluster cohesion and inter-cluster separation, helpful in evaluating whether imputation preserves biological grouping.^43^^,^^44^ Values closer to 1 imply well-separated and compact clusters, indicating that the imputed data maintain meaningful biological distinctions.
2. Calinski-Harabasz index: The Calinski-Harabasz index measured the ratio of between-cluster dispersion to within-cluster dispersion, reflecting the quality of clustering after imputation.^45^^,^^46^ Higher index values denote more distinct and compact cluster structures, suggesting that the imputation process retains or enhances the fidelity of data grouping.
3. Normalized Mutual Information (NMI): NMI assessed the agreement between the biological annotation and post-imputed clusters computed by each method. NMI ranges from 0 to 1, with higher values indicating better agreement between two classification labels.
4. Adjusted Rand Index (ARI): ARI measures the similarity between clustering assignments and reference labels while correcting for chance, providing a more robust assessment of how well imputation preserves clustering structure. ARI ranges from -1 to 1, where ARI values indicate more similar clustering results between the inferred clusters and the ground truth.^47^

#### Ranking for evaluation indexes

To evaluate imputation performance, we computed nine objective metrics, including NRMSE, MAE, RMSE, the Pearson correlation coefficient, cosine similarity, the Silhouette coefficient, the Calinski-Harabasz index, ARI, and NMI. For each metric, we used the median value across datasets to represent method-level performance. We then applied min-max scaling to the median scores of each metric to map them onto the [0,1] range for comparison on a common scale. Notably, because lower values of NRMSE, MAE, and RMSE indicated better performance, we reversed their normalized scores by computing *1- (min-max scaled value)*, so that higher values consistently indicated better performance across all nine metrics.

To derive overall rankings within different evaluation dimensions, we further computed average ranks within each category. For the imputation accuracy dimension, under both MCAR and MNAR mechanisms, we averaged the normalized scores for each metric, including NRMSE, MAE, RMSE, the Pearson correlation coefficient, and cosine similarity, and ranked the five metrics accordingly. We then averaged the resulting ranks across the five metrics to obtain the overall ranking for imputation accuracy. For the spatial clustering dimension, we ranked methods for each of four metrics based on their normalized scores, including the Silhouette coefficient, the Calinski-Harabasz index, ARI, and NMI, and we averaged the ranks across these four metrics to obtain the final ranking for spatial clustering performance. The average ranks in these two dimensions reflected the overall performance of each method in reconstructing metabolite intensities accurately and preserving biologically meaningful spatial clustering structures, respectively.

#### Evaluation of scalability

To minimize potential confounding factors, we selected two representative datasets for scalability analysis, with increasing numbers of spatial spots and metabolites. The first dataset contained 7,163 spatial spots and 116 metabolites, whereas the second dataset contained 12,595 spatial spots and 135 metabolites. Runtime was measured using *Sys.time()* in R and *time()* in Python, and if a task did not finish within the predefined time limit, it was considered to have produced no result under that setting. Peak memory usage was obtained by monitoring the maximum resident set size, and memory tracking for the R implementations was performed using the *psutil* library.

#### Statistical analysis

Statistical analyses and visualization results were conducted using R (version 4.2.0) and the Generic Diagramming Platform(https://BioGDP.com).^48^ The *ggplot2* package (version 3.4.2) and the *ggpubr* package (version 0.4.0) were used for data visualization. Detailed information, including data presentation, sample sizes, and statistical methods, is provided in relevant sections and figure legends.
