# Supplementary material for: Overcoming missing data in spatial metabolomics with machine learning imputation to accelerate downstream discovery
Source: iScience. 2026 Mar 3;29(4):115203. doi: 10.1016/j.isci.2026.115203 (PMC12999350; doi:10.1016/j.isci.2026.115203)
Supplement: Document S1. Figures S1–S9 [file mmc1.pdf]

iScience, Volume *29*

Supplemental information

Overcoming missing data in spatial metabolomics with machine learning imputation to accelerate downstream discovery

Tingze Feng, Yuhan Wang, Shaojun Pei, Qiuping Wang, Yirong Li, Jing Lv, Tian Xia, Di Chen, and Hai-long Piao

Supplemental information

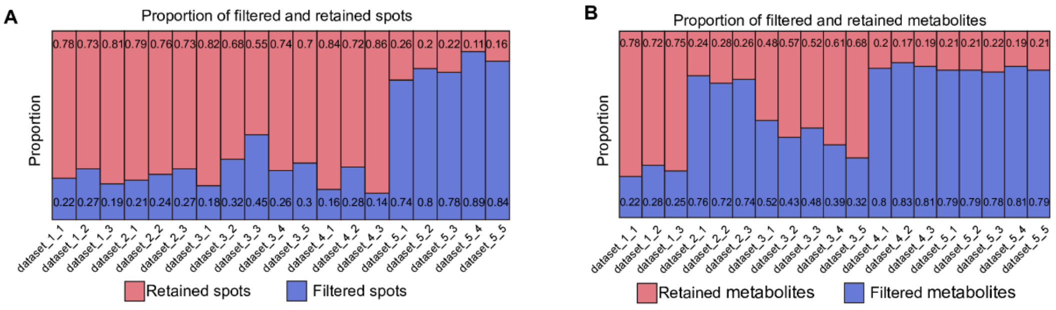

Figure S1. Bar charts of the proportion before and after filtering, related to Figure 1.

Fraction of spatial spots and metabolites retained versus filtered for imputation accuracy evaluation arm after preprocessing (removal of spots with zero intensity across all metabolites and metabolites with a missing level of more than 10%).

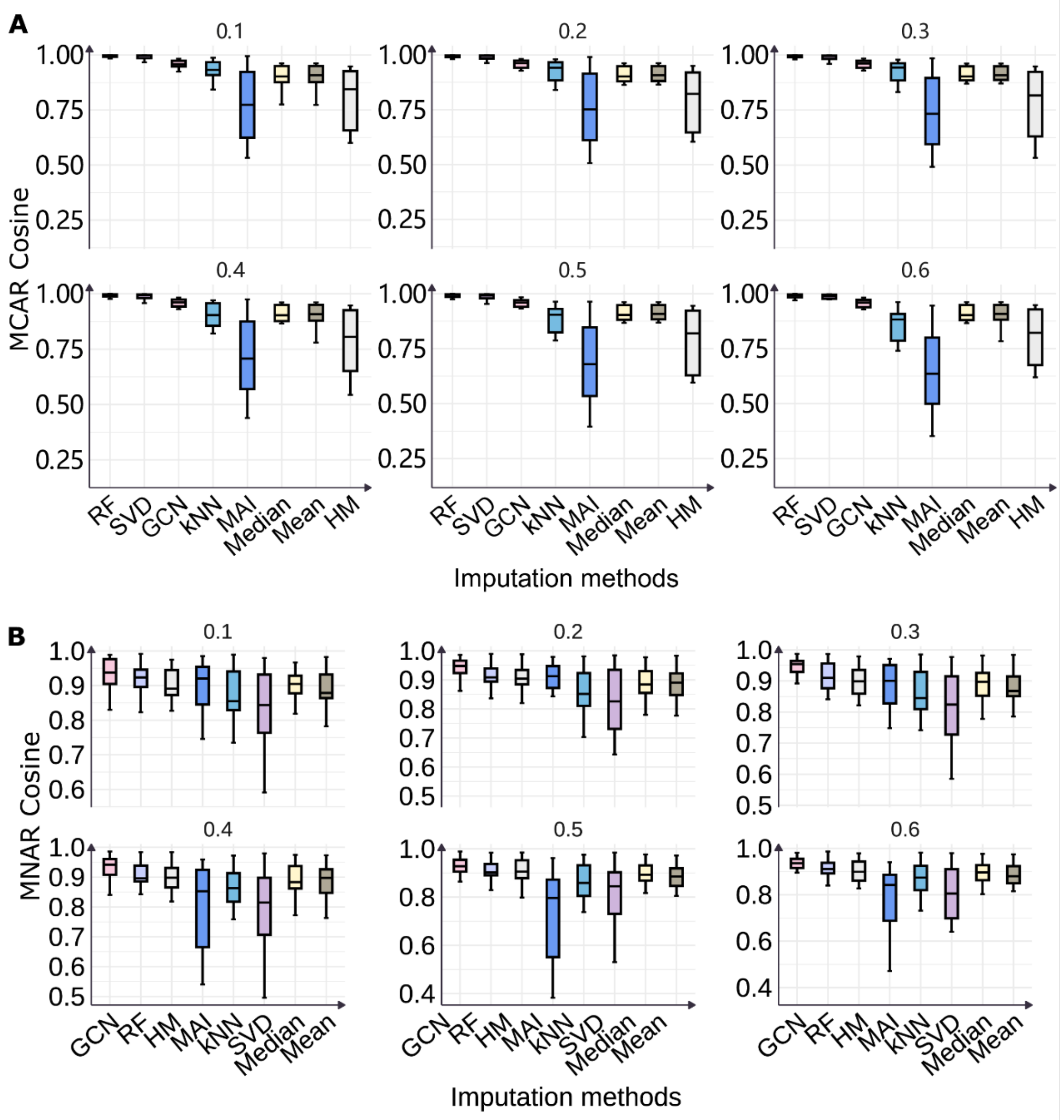

Figure S2. Boxplots display the distribution of Cosine across the 19 slices for eight algorithms, related to Figure 2.

(A) Cosine similarity stratified by dropout level under the MCAR mechanism.

(B) Cosine similarity stratified by dropout level under the MNAR mechanism.

In the boxplots, the central line indicates the median; the box boundaries represent the first and third quartiles (Q1 and Q3), and the whiskers extend to the most extreme data points within 1.5 × the IQR.

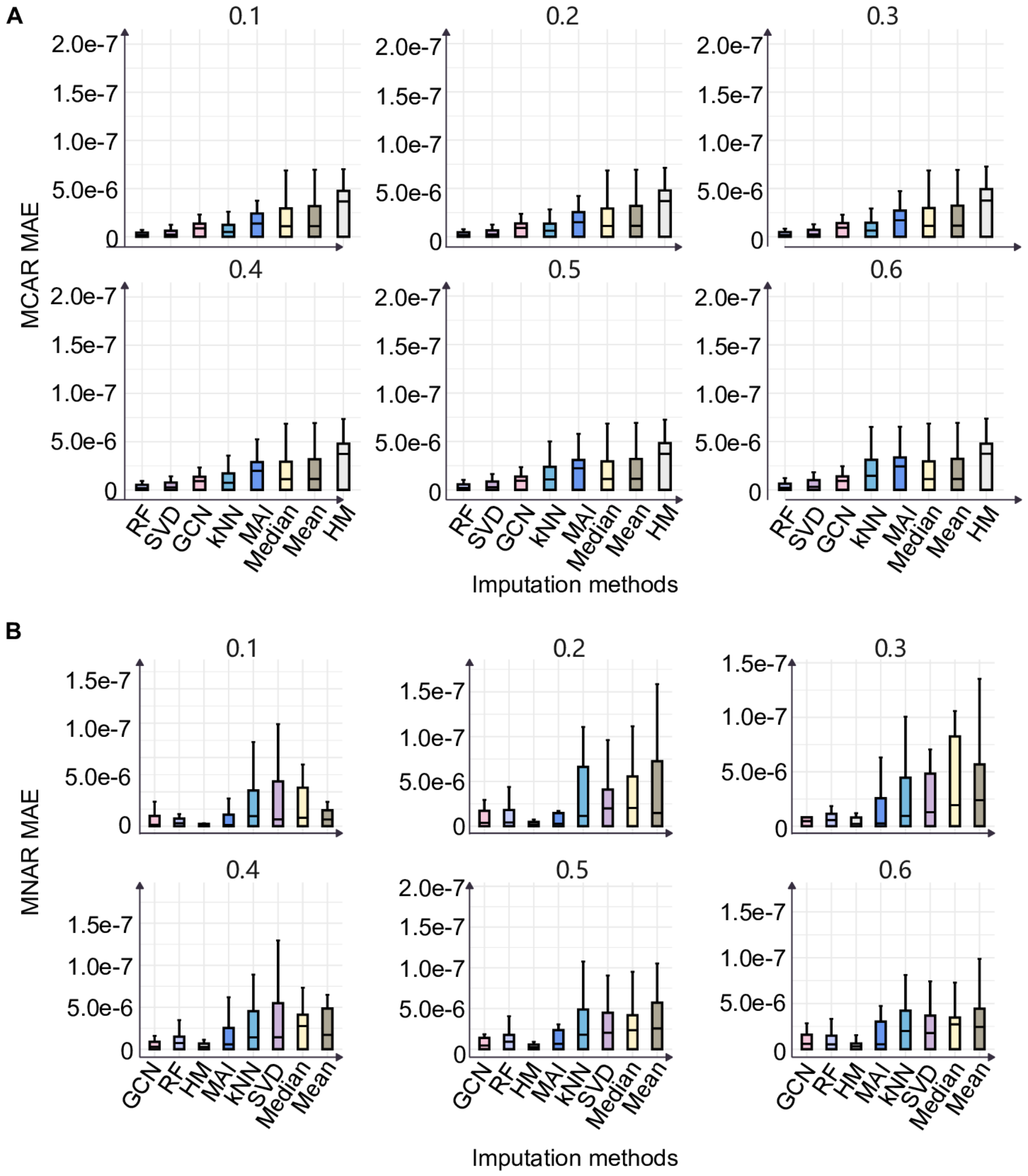

Figure S3. Boxplots display the distribution of MAE across the 19 slices for eight algorithms, related to Figure 2.

(A) MAE stratified by dropout level under the MCAR mechanism.

(B) MAE stratified by dropout level under the MNAR mechanism.

In the boxplots, the central line indicates the median; the box boundaries represent the first and third quartiles (Q1 and Q3), and the whiskers extend to the most extreme data points within 1.5 × the IQR.

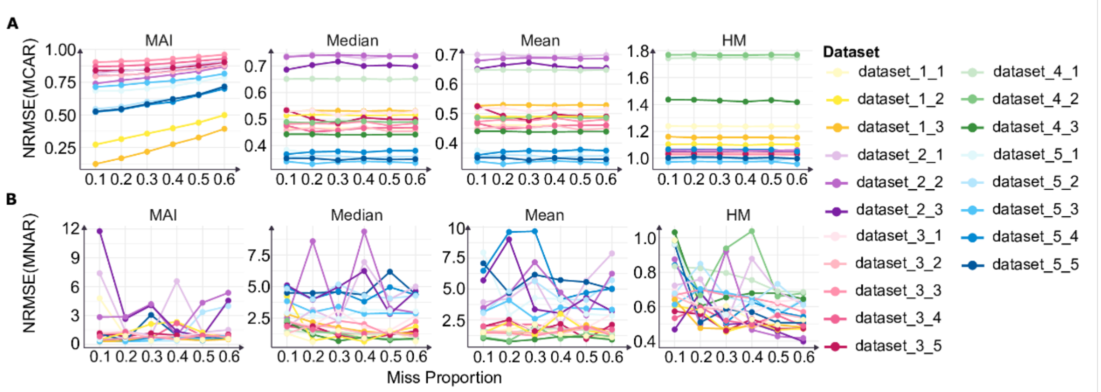

Figure S4. Line plots of NRMSE values of representative algorithms across datasets, related to Figure 2.

(A-B) MCAR (A) and MNAR (B); curves depict NRMSE as a function of dropout proportion for MAI, Median, Mean, and HM across the 19 slices, highlighting between-dataset heterogeneity and each algorithm's sensitivity to increasing missingness.

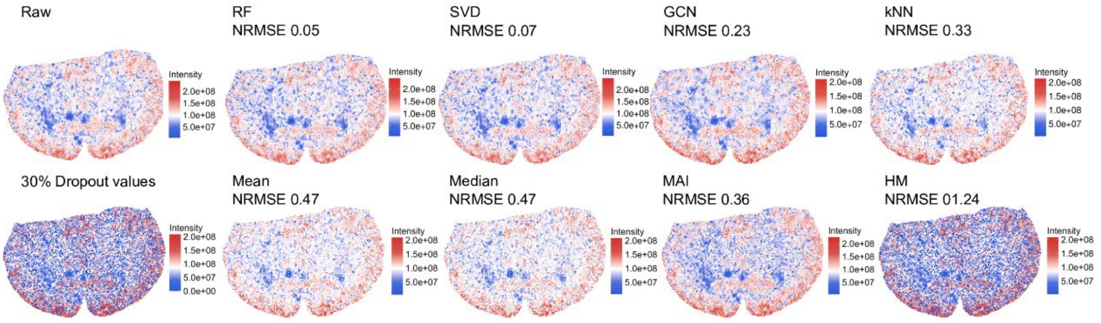

Figure S5. Spatial reconstruction example under MCAR, related to Figure 2.

Using metabolite C45H78NO7PNa in Dataset_1_1 as an example, we show the original spatial intensity map (RAW), the observed map after 30% dropout, and the imputed reconstructions from eight algorithms; color encodes ion intensity, and all images use a unified color scale derived from the raw intensity range of this metabolite to facilitate comparison; the corresponding NRMSE is annotated for each reconstruction.

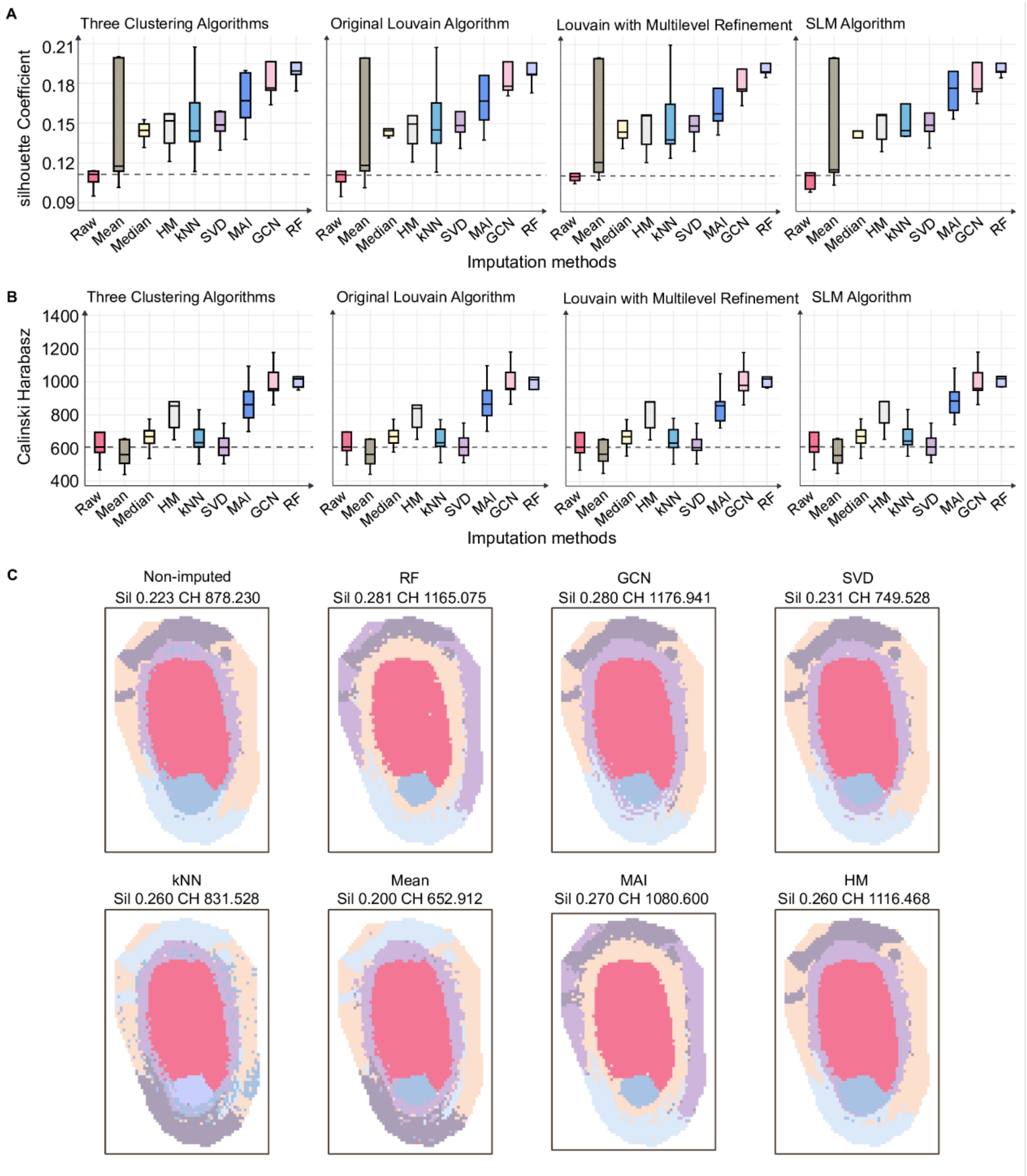

Figure S6. Evaluation of imputation effects on spatial clustering in Dataset_2_1, related to Figure 3.

(A) Silhouette coefficient across three cluster algorithms and nine resolution settings (0.1, 0.2, 0.3, 0.4, 0.5, 0.6, 0.7, 0.8, 0.9). For each imputation method, boxplots summarized calculated indexes over the nine resolutions; the dashed horizontal line indicated the median index of the raw (non-imputed) baseline. The first boxplots pooled results across all three cluster algorithms and all resolutions; the subsequent three boxplots report, for each clustering algorithm separately, the distribution of scores across the nine resolutions. Higher values indicated more compact and well-separated clusters.

(B) Calinski-Harabasz index evaluated under the same design as in (A): boxplots over the nine resolutions per method, points for individual resolutions (0.1-0.9), and a dashed line marking the raw-data median reference. Higher values indicate stronger between-cluster separation relative to within-cluster dispersion.

In the boxplots (A-B), the central line indicates the median; the box boundaries represent the first and third quartiles (Q1 and Q3), and the whiskers extend to the most extreme data points within 1.5 × the IQR.

(C) Visualization of spatial clustering for raw data and seven imputed methods (algorithms labeled), shown at clustering resolution = 0.2 using the SLM cluster algorithm. Colors denote cluster identities in spatial spot coordinates. All clustering analyses were performed on pre-processed data (metabolites with > 80% missingness removed), using identical spatial coordinates downloaded from MATASPACE. The corresponding silhouette coefficient and Calinski-Harabasz index were annotated for each spatial clustering. Sil: silhouette coefficient; CH: Calinski-Harabasz.

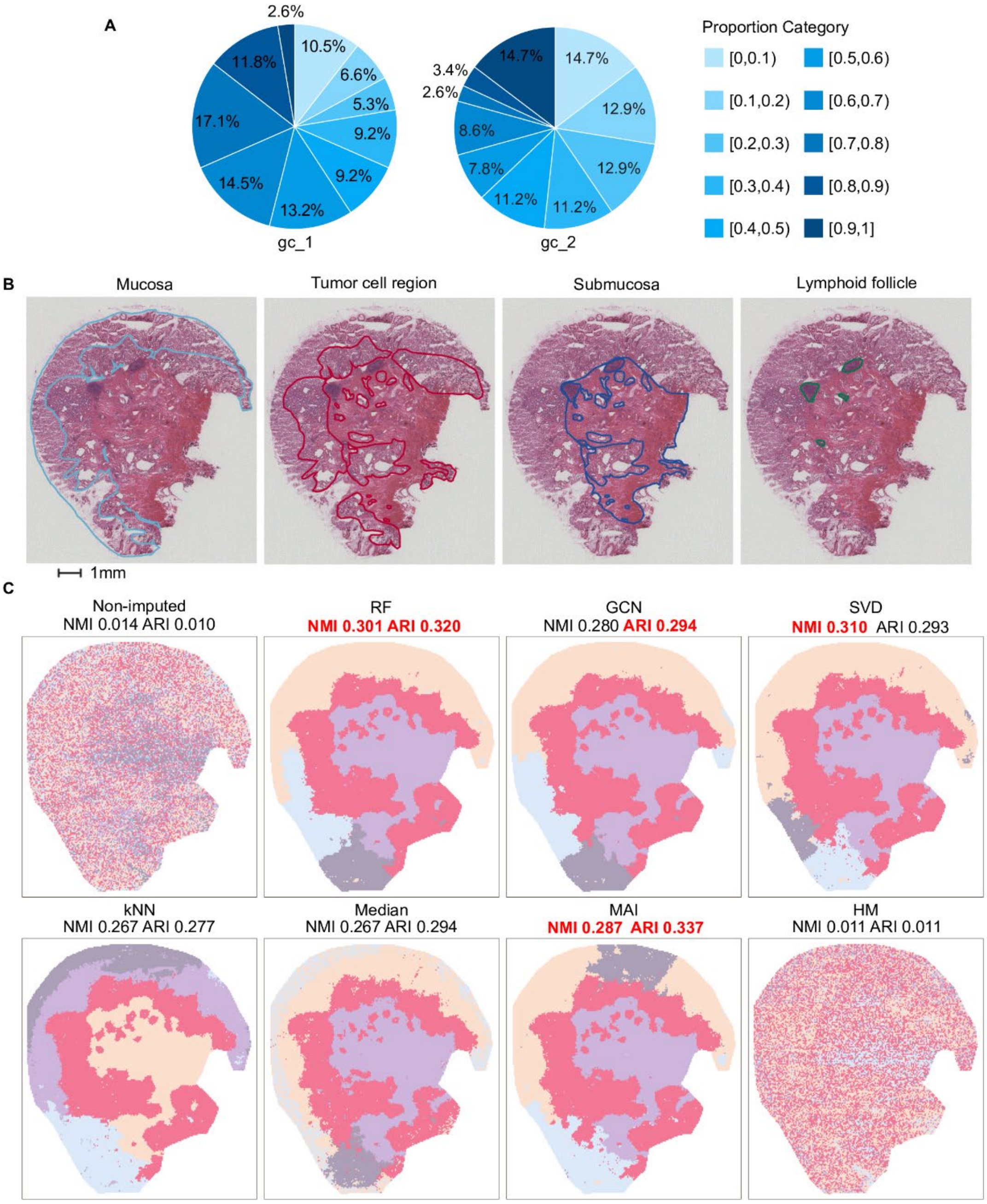

Figure S7. Extended assessment of the impact of imputation on spatial clustering in the human gastric cancer sample, related to Figure 3.

(A) The proportion of spatial spots with zero intensity in the raw spatial metabolomics data for each metabolite of two gastric cancer samples (gc_1 and gc_2).

(B) The H&E-stained tissue section with pathologist-annotated regions. The regions outlined in different colors in the figure corresponded to distinct histological areas annotated by the pathologist. Scale bar = 1 mm.

(C) Spatial clustering results obtained without imputation and after imputation using seven different methods; all methods were constrained to produce five clusters. For each spatial clustering result, the corresponding NMI and ARI values were labeled. The two values highlighted in red corresponded to metrics that ranked within the top three among the eight

cluster results.

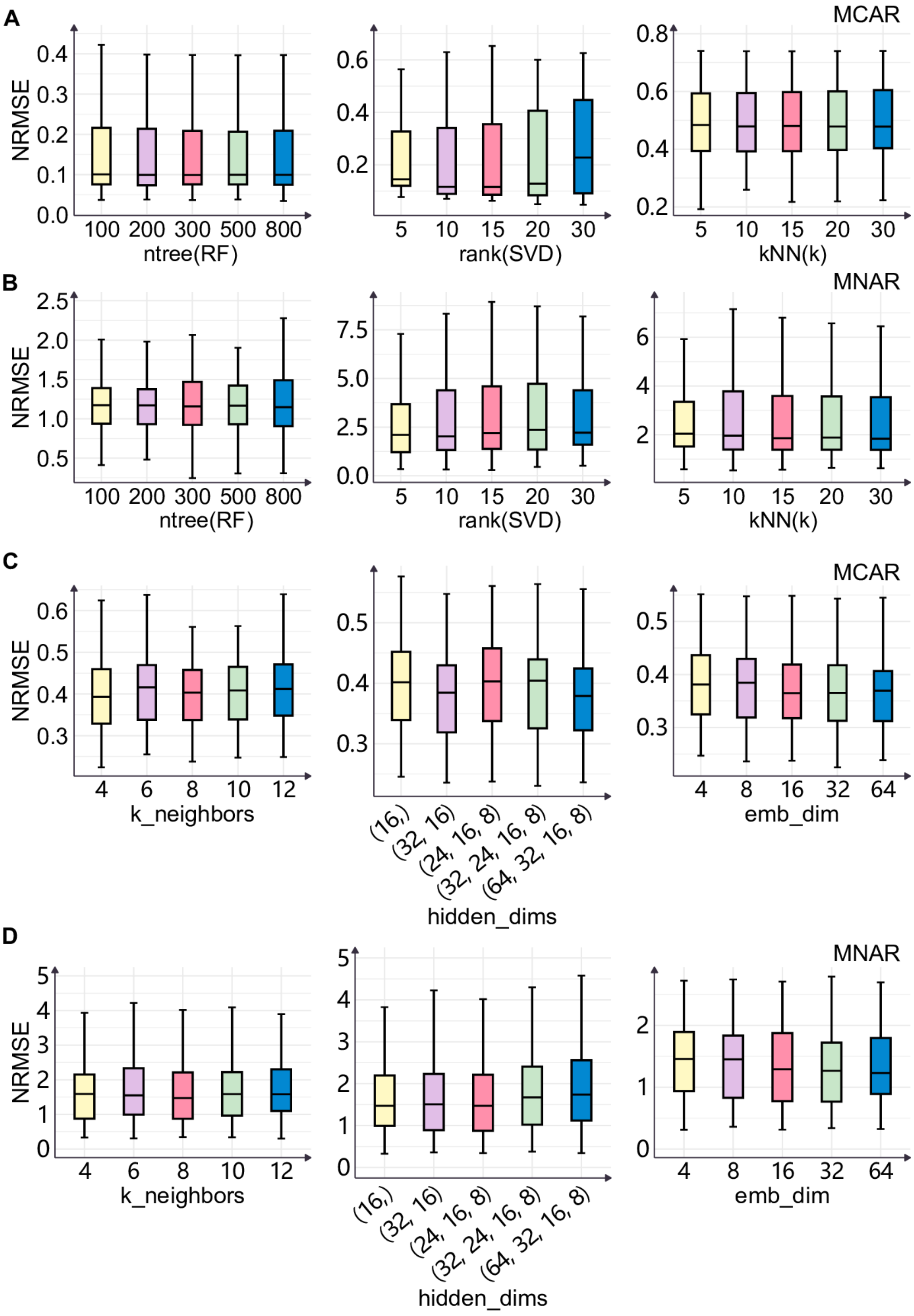

Figure S8. Sensitivity analysis of key hyperparameters for imputation algorithms, related to STAR Methods.

(A-B) Under simulated MCAR (A) and MNAR (B) conditions across 19 sub-datasets, we performed parameter tuning for RF, SVD, and kNN. Boxplots show the distribution of NRMSE across datasets for each parameter setting, including RF *ntree*, SVD *rank*, and kNN neighborhood size *k*.

(C–D) Under simulated MCAR (C) and MNAR (D) conditions, we conducted ablation-based tuning for GCN. Boxplots summarize NRMSE distributions across datasets for different graph neighborhood sizes *K* used to construct the kNN graph, different hidden_dims, and different emb_dims.

In the boxplots, the central line indicates the median; the box boundaries represent the first and third quartiles (Q1 and Q3), and the whiskers extend to the most extreme data points within 1.5 × the IQR.

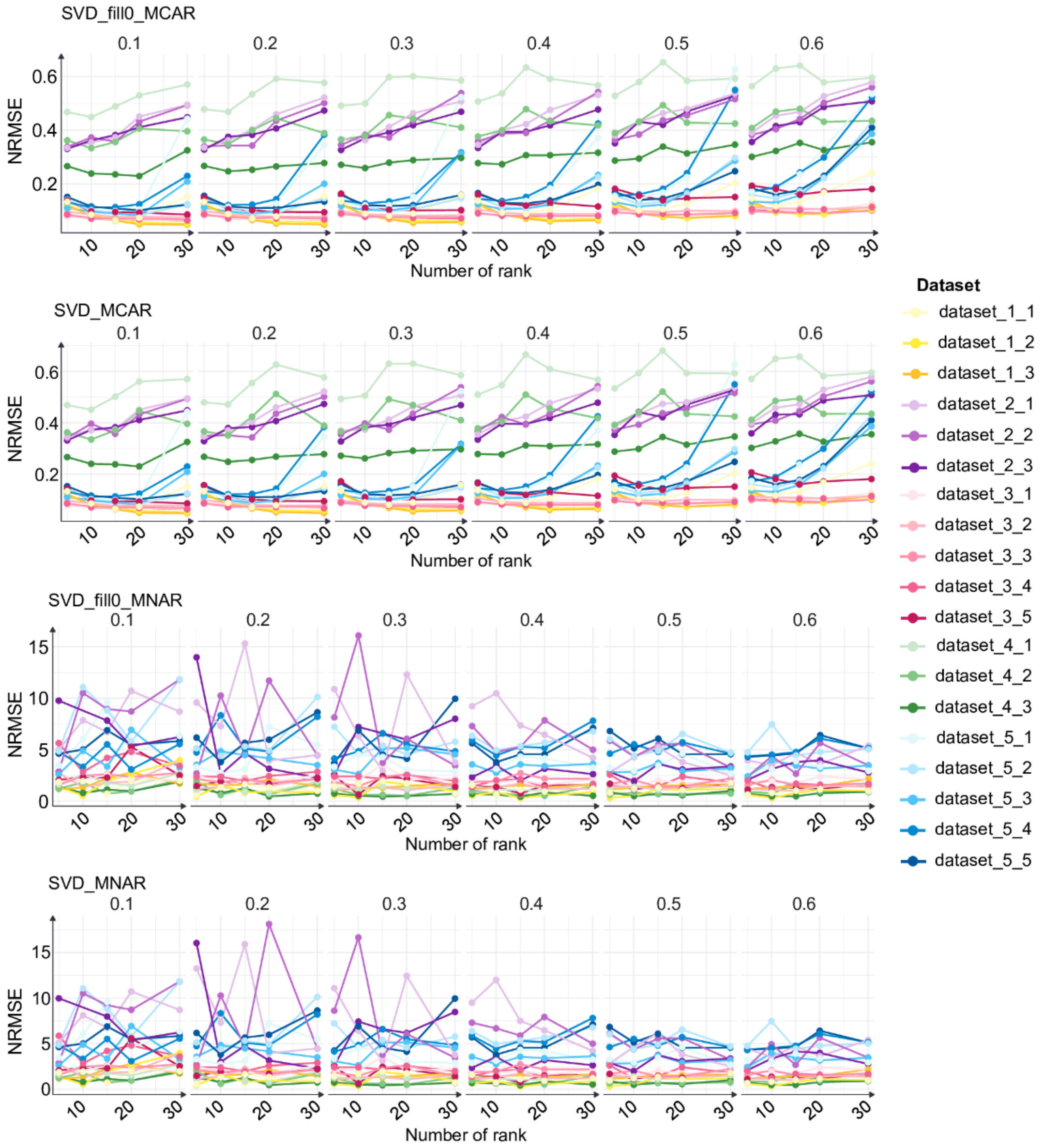

Figure S9. Impact of zero clipping on SVD imputation performance under MCAR and MNAR missingness, related to STAR Methods.

Across 19 slices, we compared SVD imputation results with and without zero clipping of negative values under simulated MCAR and MNAR mechanisms. Line plots show NRMSE trends across dropout levels for different SVD rank settings.
